# Review: Cantilever-Based Sensors for High Speed Atomic Force Microscopy

**DOI:** 10.3390/s20174784

**Published:** 2020-08-25

**Authors:** Bernard Ouma Alunda, Yong Joong Lee

**Affiliations:** 1 School of Mines and Engineering, Taita Taveta University, P.O. Box 635-80300 Voi, Kenya; benalunda10@gmail.com; 2 School of Mechanical Engineering, Kyungpook National University, Daegu 41566, Korea

**Keywords:** microcantilever, atomic force microscope, ultra-short cantilevers, high-speed atomic force microscope, biosensors

## Abstract

This review critically summarizes the recent advances of the microcantilever-based force sensors for atomic force microscope (AFM) applications. They are one the most common mechanical spring–mass systems and are extremely sensitive to changes in the resonant frequency, thus finding numerous applications especially for molecular sensing. Specifically, we comment on the latest progress in research on the deflection detection systems, fabrication, coating and functionalization of the microcantilevers and their application as bio- and chemical sensors. A trend on the recent breakthroughs on the study of biological samples using high-speed atomic force microscope is also reported in this review.

## 1. Introduction

Since its debut in 1986, atomic force microscopy has evolved from a wobbly method to one of the most utilized tool for nanoscale characterizations. The early years of atomic force microscope (AFM) use were devoted to pushing the resolution boundary, to some unimaginable extent. However, during the last decade, research has been dedicated more to force measurements, identification and characterization of processes at the molecular level, force spectroscopy, and chemical force microscopy. The atomic force microscope has the ability to detect pico-newton scale intermolecular forces using a microcantilever as a force sensor thus aiding in the investigation of intermolecular interactions between receptors and ligands in biological systems in addition to mechanics of the single living cells and biomolecules [1,2,3].

The AFM microcantilevers are not restricted to the measurement of forces and displacements accurately and precisely, but owing to their ability to be used as a spring, they can be used as a motion sensor to detect nanoscale vibrations of various prokaryotic and eukaryotic cells [4]. From single-molecule to single-cell manipulation, the AFM became a multifunctional toolbox for observing and measuring various biophysical parameters of cellular and subcellular assemblies and machineries [5,6].

In addition to microcantilevers, microfabricated devices of different geometries such as flat pattern, micro-fluidic devices, micropillars, and microwells have been developed, and they are used to study the forces generated by various cells. The beauty of the micropillars, for example, is that they can be fabricated to the submicron range by using several nanobased techniques that include molding and lithography. In addition, the methods provide an easy way to vary the geometric parameters. Thus, the micropillars provide a versatile platform on which various cells can be examined [7,8]. Furthermore, the micropillars can be easily modified like the microcantilevers so that the cells are only attracted to the top surface only, while the remaining parts are covered with a repelling hydrogel layer. The micropillar-based sensors are promising tools for the measurements of biological samples in addition to other sensing applications including the flow of fluids and shear stresses [9]. The microchannel devices have also been developed as sensors for biological and chemical applications. These sensors are used for various biosensing applications because they can allow parallel processing of numerous samples within the same chip. It has been reported that microchannel biosensors can not only increase the detection sensitivity but also decrease the cost when compared to the conventional detection methods [10]. Other advantages of the microchannels-based sensors include real-time detection, high throughput, enhanced analytical performance, and portability. Moreover, it is possible to analyze most biomolecules in their solutions so as to imitate the natural environment close to in vivo. Thus, microchannel devices have become the most suitable methods for the development of some specific biosensors. Microchannels have critical length dimensions in the range of 1 to 100 mm and are characterized by a high surface area to volume ratio. The main limitation of microchannel biosensors is that most of the techniques involved in the fabrication of these devices are only capable of creating features with specific geometries. It is not possible to mimic those in their natural vasculature. The sensors, however, are reported to exhibit numerous excellent characteristics including low cost, portability, high sensitivity, and simple instrumentation [11]. Mi et al. [12] reported an amperometric lactate biosensor based on electrodes modified by Prussian blue. They immobilized the lactate oxidase enzyme using chitosan–carbon nanotubes. The biosensor was integrated with flow microchannels, and they were able to achieve a high sensitivity of 567 nA mM^−1^ mm^−2^.

The atomic force microscope has several advantages when compared to other microscopic surface characterization techniques, such as optical fluorescent microscopy (OFM), optical confocal laser scanning microscopy (OCLSM), transmission electron microscopy (TEM), and scanning electron microscopy (SEM). Quantifiable and accurate surface height information, down to the sub-nanometer scale level, is attainable by using the atomic force microscope. On the contrary, OFM, OCLSL, SEM, or TEM cannot provide three-dimensional topographies. In addition, the AFM can permit imaging in air, aqueous, or even under vacuum conditions over a wide range of temperatures. The feasibility of observing the samples in liquid media at room temperature [13] and the capability of scanning an area of interest from the nanometer to the sub-millimeter scale open the possibility of studying many systems under physiological conditions from the macro level to cells and tissues [14] at an unrivaled resolution. Furthermore, the sample preparations are considerably easier compared to the TEM or SEM. Researchers can take advantage of the simple sample preparation for AFM, which allows studying living samples through surface imaging and mechanical mapping at the same time. For example, in cancerology, the AFM has been extensively used as an innovative diagnostic tool to explore the effects of cytotoxic drugs [15]. With a relatively simple setup and principle, AFM can probe the tissue dynamics at the nano-scale. After image acquisitions using the AFM, other surface mechanical/electrical/magnetic property characterizations can be performed in both quantitative and qualitative manners [16].

Compared to AFM capable of observing high-resolution of cellular processes in their native environments, the electron microscopy methods such as scanning electron microscope (SEM) and transmission electron microscope (TEM) can equally achieve nanometer level resolution but are limited in several aspects [17]. They require extensive sample preparations owing to the high-vacuum conditions required for the operations and limited sampling speed for possible real-time observations [18]. Furthermore, the traditional electron microscopes only allow imaging of samples in the unhydrated state. Even with the development of the environmental electron microscope, it is still not possible to image in a perfect liquid environment [19]. Other researchers have also reported potential damaging of cells by the electron irradiations [20]. The damages may include breakage of the molecular bonds, death of the cells, and generation of the reactive solvate electrons [21]. However, it has been recently reported that there is a possibility of employing electron microscope for the study of live bacterial cells if the radiation dosage is a few orders above the lethal dose needed to cause reproductive cell death [22].

**Figure 1:**
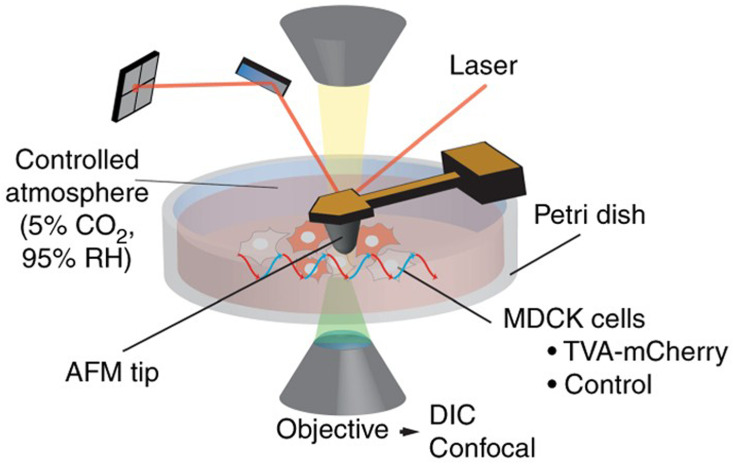
Combined force distance-based atomic force microscope and confocal microscopy for life science used to study the binding effects of a single virus onto the surface of a mammalian cell. Reprinted from the work in [24].

AFM provides a technology that can also be integrated with other microscopic and spectroscopic techniques such as laser scanning confocal microscopy (LSCM) [23,24], total internal reflection fluorescence microscopy (TIRFM) [25,26,27], aperture correction microscopy (ACM) [28], correlative stimulated emission depletion microscopy (STEDM) [29,30,31], fluorescence lifetime imaging microscopy (FLIM) [32,33], stochastic optical reconstruction microscopy (STORM) [34,35], super-resolution fluorescence microscopy (SRFM) [36], tip-enhanced raman spectroscopy (TERS) [37,38,39], scanning near-field optical microscopy (SNOM) [40,41,42], and Förster resonance energy transfer (FRET) [43]. These correlative approaches offer a good spatial (nm) and high temporal (ms) resolution to study cellular and molecular biophysics. For example, Newton and his co-workers [24] developed a novel approach for quantifying the binding events of a single virus onto the surface receptors of a mammalian cell surface. They integrated a force–distance-based atomic force microscopy and fluorescence microscopy operated from below. By using the functionalized microcantilevers, they were able to gain an insight into the initial binding effects of a single pseudo-typed rabies virus to the avian tumor virus cells receptors artificially expressed in MDCK cells. A schematic of their set-up in Figure 1 shows the functionalized microcantilever using amine groups probing the MDCK cells with TVA950 receptors.

The microcantilever is the heart of the atomic force microscope and its existence can be associated with the invention of the atomic force microscope over three (3) decades ago [44]. The microcantilever sensor has a sharp probe attached to the free end. With the presence of a cantilever probe, the amplitudes, phases, and frequencies of various modes of resonance can be utilized. Moreover, the AFM can create nanoscale patterns with lithographic techniques using a conductive probe or obtain mapping of sample chemical identity when combined with optical spectroscopy techniques. They have attracted tremendous attention, and numerous biological [45], biomedical [46,47], physical, and chemical [48,49,50,51] applications have been demonstrated.

For biological applications, the microcantilever biosensors should be sensitive, fast, and flexible for identification of biomolecules and high-throughput screening in the pharmaceutical industries [52]. They have also been applied in the study of biosample stiffness measurements [53], surface morphological and mechanical analysis [54], and viscosity–density sensing in liquid media.

For physical detection, Markidou and his co-workers [55] developed a sensor that can measure the shear and elastic modulus of a soft material using a piezoelectric microcantilever. The microcantilever consisted of a highly piezoelectric material with a stainless steel material coating. Thus, in response to an applied voltage, the material bends creating an axial force capable of generating stress on the soft material. Other physical parameter detection measurements that have been conducted using microcantilevers include viscosity measurements, thermal analysis in picoliter solid samples, and detection and identification of trace amounts of biological species using a combination of micro-calorimetric spectroscopy and microcantilever thermal detectors [56,57,58,59].

The ability of microcantilevers to change their vibrational frequencies or levels of deflection upon adsorbing molecules on their surface makes them excellent probes that can act as chemical, physical, or biological sensors at the nanoscale. Changes in the vibrational frequency of micromechanical devices can be used to measure viscosity, density, and flow rates in various systems. Deflections of the cantilever are due to the stress from the molecular adsorption, which can be upward or downward depending on the type of chemical bonding of the molecule. In these systems, the change in the frequency of a microcantilever has been reported to be proportional to the magnitude of the adsorbed mass [48,60,61,62]. By using this phenomenon, the microcantilevers have been employed in the measurement of various physical phenomena such as humidity [63,64,65,66,67], temperature [68,69], and pressure [70,71]. For temperature sensing, the deflection of a silicon nitride microcantilever coated with a different material such as gold or aluminum is monitored. When there is a temperature change, the microcantilever bends due to the differences in the coefficients of thermal expansions of two materials [72,73]. Such sensors are found to exhibit a linear relationship between the deflection of the microcantilever and the changes in temperature as opposed to the thermal resistance or bi-metallic temperature sensors. The thermal resistance temperature sensors possess high thermal nonlinearity whereas the bi-metallic temperature sensors have a slow response and low resolution.

For humidity sensing, the microcantilevers can be coated with a water absorbing polymer [74]. For example, Singamaneni and his co-workers achieved a sensitive humidity sensor by coating a flexible silicon nitride microcantilever with a plasma-polymerized methacrylonitrile monolayer [75]. A linear relationship was observed as a function of time for both humidification and desiccation and a fast response time. Often, the microcantilever chemically or physically interacts with the environment resulting in an increase in the mass of the cantilever thus decreasing the resonant frequency. Sometimes the chemical reaction can cause an increase in the stiffness of the material. Their beauty, however, lies in their miniature structures, simplicity, and the possibility of mass production with good reproducibility [49].

In gas detection, for example, the gas molecules in the area surrounding the sensor are selectively adsorbed leading to an increase in the mass of the microcantilever that causes a proportional shift in the vibrational frequency according to the mass of the adsorbed gases. Another variable that can be used to quantify the amount of the adsorbed gases in the microcantilever is the change in the resonant frequency caused by the surface stresses. However, when compared to the mass loading, the effects of such stresses on the resonant frequency are found to be insignificant [76,77].

In this review, we look at the recent developments in microcantilever-based sensors in atomic force microscopy, latest improvements in various methods of microcantilever excitations for atomic force microscopes, progress in microcantilever fabrication, and modification suitable for biosensors or chemical sensors. We also address the progress in the development of ultrashort-microcantilevers and high-speed atomic force microscopy and their application to the study of life sciences.

## 2. Generic Operations of the Microcantilever

**Figure 2:**
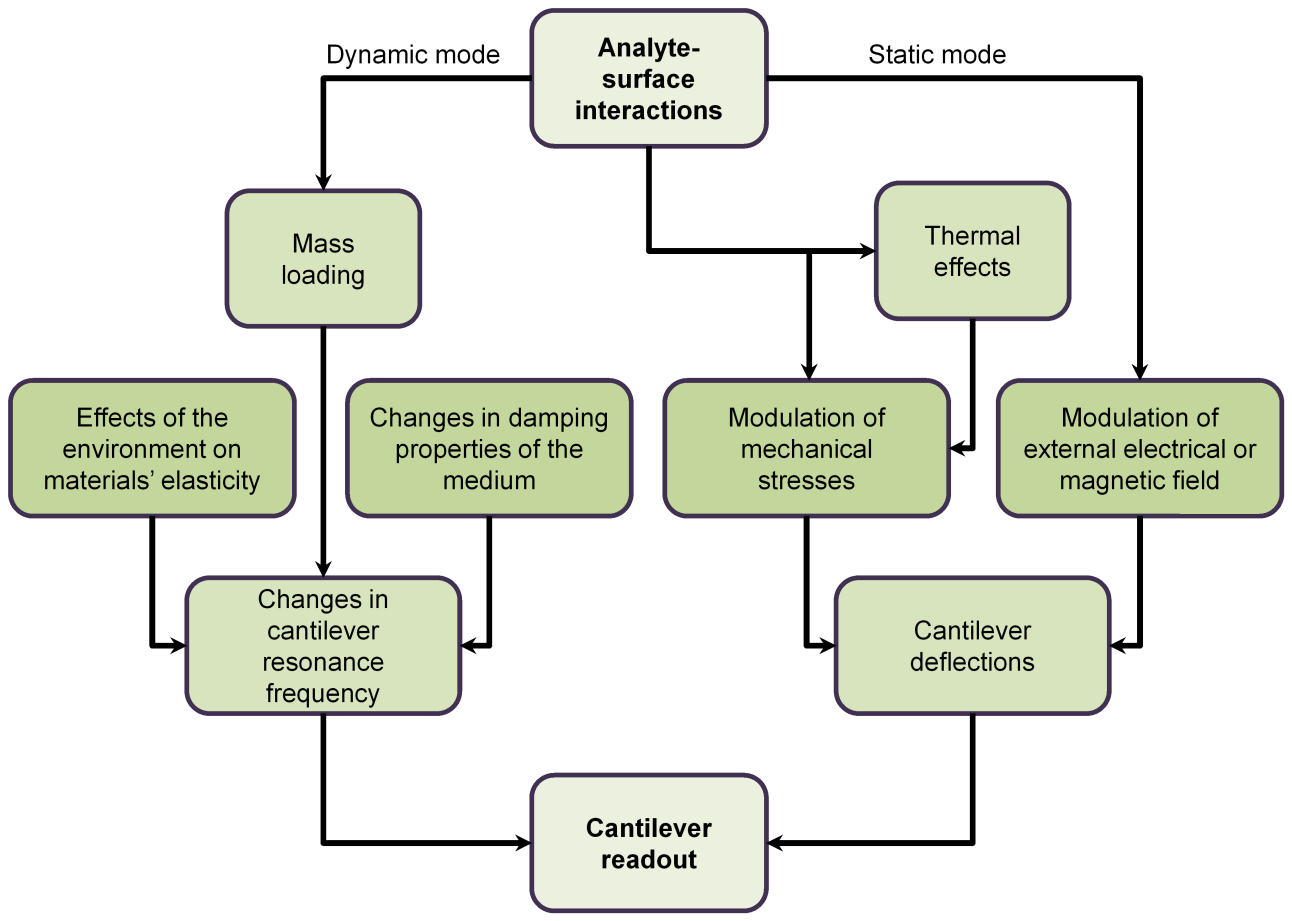
A flowchart describing the modes of operation and the principle of transduction for the microcantilevers. Adapted from the work in [80].

There are basically two modes of operations of the microcantilevers: static mode and dynamic mode. In the static mode, the microcantilever remains stationary, and its deflections only depend on surface stress variation. However in the dynamic mode, the microcantilever is externally actuated to oscillate about its natural resonant frequency. Thus, it is possible to accurately determine any mass change caused by adsorption of molecular layers. Typically, either the upper or both the upper and the bottom surfaces of the cantilever are coated with an active film followed by the close observations of the changes in the resonant frequencies or the quality factor caused by an addition of mass on the sensor [78,79]. The general overview of the operations of the microcantilevers is shown in Figure 2.

The microcantilever systems are simple mechanical devices with the dimension in the micrometer regime. Often, for ease of handling, the devices are attached at one end to a chip or support. Compared to other conventional sensors, microcantilevers are often preferred sensors due to the numerous advantages such as high precision, high sensitivity, rapid response, large dynamic response, miniaturization, high reliability, and large-scale integration [81,82,83]. Most importantly, these sensors can be microfabricated and mass produced [84], greatly lowering their costs. The wide use of microcantilever sensors has been contributed to their high sensitivity. The high sensitivity to surface phenomena is contributed by their large surface area-to-mass ratios [85]. Due to their high sensitivities, the response characteristics of the microcantilevers such as phase, amplitude of deflection, frequency changes, and quality factors can be easily detected using either electrical or optical detection means. Thus, it is possible to measure small forces in the pico-newton regime with relative ease. Some of the areas of applications are outlined in Table 1.

## 3. Modes of Operations of Cantilever-Based AFM

**Figure 3:**
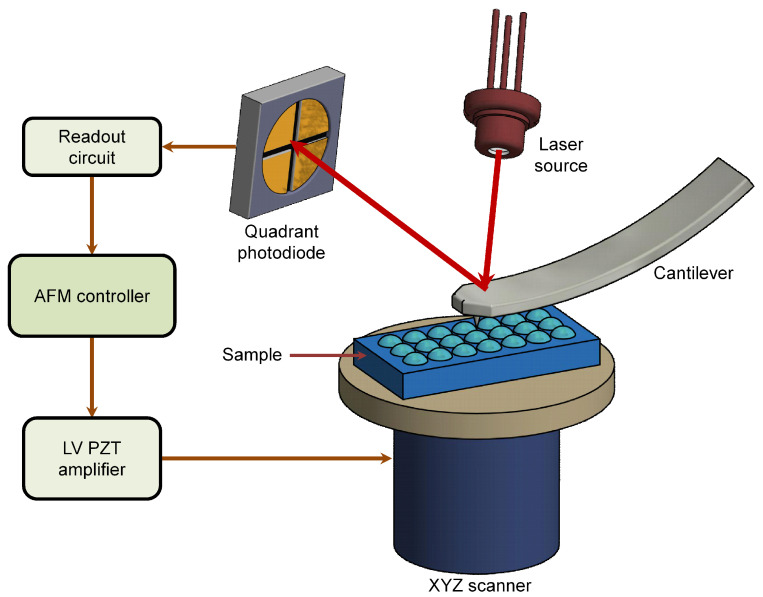
General schematic of a cantilever-based atomic force microscope (AFM) with the laser reflecting onto the photodetector.

The AFM typically consists of several components including: scanner, a cantilever with a sharp probe, a light source, an electronic feedback controller used to maintain a given set-point and a position sensitive photodetector (PSPD). Figure 3 shows a classic example of an AFM setup. A flexible cantilever typically fabricated from silicon or silicon nitride with a sharp tip at the free end is brought into close proximity with the sample (several angstroms) where it interacts with the sample surface due to the existence of the Van der Waal’s forces. This interaction force, usually in the nano-newton (nN) range, causes a deflection of the flexible cantilever. The PSPD monitors and measures the amount of deflection of the flexible cantilever in proportion to the strength of the interactions. A transducer, usually piezoelectric stack actuator enables positioning of sample in the lateral direction and the cantilever probe in the out-of-plane direction with very precise motions. The AFM controller through an electronic feedback loop is then used to regulate the tip-sample interaction and to maintain a constant separation between the tip and the sample. The output of this feedback loop can be used to obtain topographical information. This simple instrument has turned out to be one of the most powerful tool that allows visualization of objects at nanoscale.

Different microscopes that are used to extract information about a sample surface have different “modes” of operations. For example, the backscattered or secondary electrons in scanning electron microscopes are utilized in order to image and provide information about topography and chemical compositions, respectively. On the other hand, the optical microscopes can operate in a polarized, dark-field, bright-field, or phase contrast mode, depending on the optical elements used in its operation. Similarly, the atomic force microscope can operate in a number of different modes. A few of the modes are highlighted in this section. The various operational modes are derived from different methods of exciting a cantilever or whether the microcantilever is in contact with the sample surface or not while in operation.

**Figure 4:**
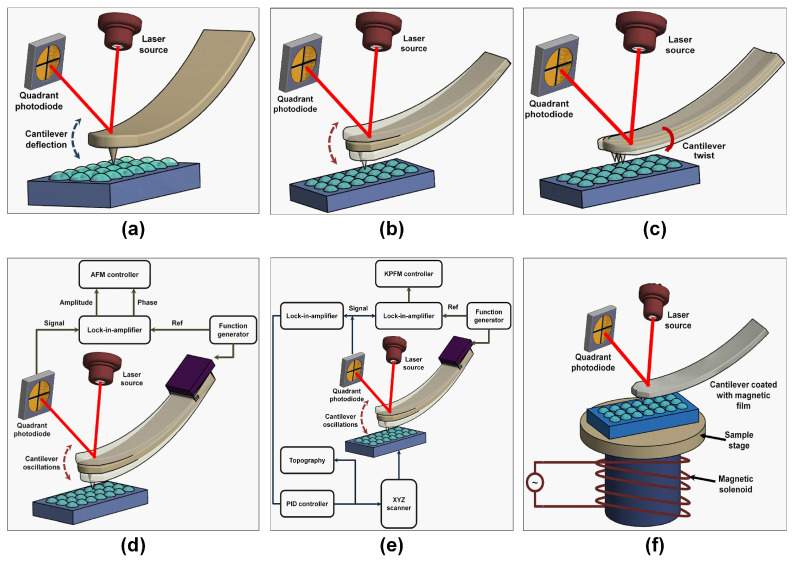
The AFM working modes: (**a**) contact mode, (**b**) tapping/dynamic mode, (**c**) frictional force microscopy, (**d**) phase mode, (**e**) Kelvin probe force microscopy, and (**f**) magnetic force microscopy.

Contact mode (CM) is the mother of all the imaging modes and still the most popular mode frequently used in many commercial atomic force microscopes [102]. Here, a sharp probe on the lower side of the micro-machined cantilever is constantly in contact with the sample surface as shown in Figure 4a. Therefore, the interaction between the cantilever and the sample is repulsive. Any variations in the sample topography are detected using an integrated optical sensor [103,104,105,106] that senses the deflection in the micro-machined cantilever owing to the variation in the interaction forces between the sharp tip and the sample. Significant frictional forces are generated when the cantilever raster scans the sample surface due to the applied force in the vertical direction. In contact mode, the operator is able to track stiffer and rougher surfaces better at higher scan speeds [107].

Non-contact mode (NCM) AFM has transformed the field of atomic force microscopy, thanks to Martin [108] and his research group who pioneered the technique just a year after the AFM was discovered. NCM operates in the attractive regime of the force–distance curve. Concisely, the cantilever together with a attached sharp probe oscillates above the sample surface at a preset scan speed [109,110] as shown in Figure 4b. During the scanning operation, the tip and sample distance should be maintained constant for the entire scanning period. This is made possible by tracking the changes in the phase, amplitude, or frequency of the cantilever induced as a result of the attractive forces (pico-Newton range). This interaction force is used in the feedback loop [108,111]. The small interaction forces offer the ability to image soft samples without damaging them. Furthermore, unless the tip crashes onto the sample surface, the probe remains undamaged and sharp during the whole scanning operation thereby increasing the operational lifetime of the probe. However, NCM usually has a limitation of slower scan speeds than contact mode in order to remedy the adsorbed fluid layer which is sometimes excessively thick to guarantee effective measurements [112]. This mode is not frequently used for biological sample characterization.

Friction force microscopy (FFM) is a form of static mode (contact mode). Here, the microcantilever tip and the sample surface is brought into repulsive contact. The FFM mode is often used for measuring the friction of a surface as the cantilever twists side to side by a torque, measured as the probe raster scans along the sample surface. The torsional changes are simultaneously recorded using the photodetector as a twisting of the cantilever together with the topographic features measured as a normal bending as shown in Figure 4c. FFM is commonly used to obtain a qualitative frictional contrast of the surface. However, the surface frictional coefficient can be calculated with an appropriate calibration of the lateral cantilever spring constant.

During the tapping mode operation of the AFM, simultaneous phase and topographical images can be acquired. In phase imaging (PI), the system monitors the phase lag between the signal that drives the cantilever oscillation and its output signal (see Figure 4d). Phase images can be used for assessing the information about the composition, adhesion, and viscoelastic properties of a sample surface.

Kelvin probe force microscopy (KPFM) was first recorded about three decades ago and enables imaging of surface potential at nanoscale for a variety of materials. In this technique, a contact potential difference is measured between a conductive AFM probe and the sample of interest. In KPFM, an external bias is applied to negate the contact potential difference while monitoring the cantilever amplitude at the resonant frequency (see Figure 4e).

When a microcantilever is coated with a magnetic material, the AFM can be used to study magnetic domain structures of a surface with high resolution up to 10 nm depending on the sharpness of the probe. The magnetized oscillating sharp probe first scans the surface to get topographical information, followed by an elevation of the probe off the surface by a set distance and recording the long range magnetic interaction force resulting in the magnetic force microscopy (MFM). A set-up showing the basic principle of MFM is shown in Figure 4f. In addition to magnetic forces, Van der Waals forces also act on the sample however they are weaker in magnitude. Thus, in MFM, the Van der Waals forces can be tapped to obtain the topography of the samples.

In the mechanical mapping mode (MMM), the AFM measures the sample stiffness, in terms of Young’s modulus values, through a nanoindentation technique. In AFM nanoindentation, the AFM collects indentation-force curves on the sample of interest. The obtained indentation–force curves can be fitted using linear elastic contact mechanical models, such as the Hertz model, in order to estimate Young’s modulus. However, the technique can be very slow with one indentation per second taking over 60 min for single modulus mapping. Significant improvement in this aspect has been achieved by some AFM manufacturers now able to acquire high resolution force maps within a few minutes. At such a high acquisition speed, however, indentations of a viscoelastic time-dependent materials may lack accuracy. In addition, the tip radius, which is vitally important for the modulus calculation, will be increasingly inaccurate during the course of imaging.

Another imaging mode that has been developed for materials with high elastic modulus is the contact resonance imaging (CRI). In CRI mode, the sample is oscillated at the resonance frequency while the microcantilever tip is in contact with the sample. Mostly, the technique has been applied in the study of biological materials. CRI can provide information about the nano-mechanical properties from very small volumes. Moreover, the fact that CRI can measure viscous as well as elastic properties of materials makes it a suitable tool for studying composite materials.

In the multifrequency force microscopy (MFFM), multiple cantilever frequencies (higher harmonics and/or higher flexural eigenmodes) are excited to provide information about the tip-sample nonlinearities are recorded [113,114]. With MFFM, there is a potential of overcoming many hurdles including high throughput, material properties, and spatial resolution. Multiharmonic mode uses changes in the amplitude, the phase of the oscillator, and other appropriate harmonics in order to offer quantitative local property maps [115]. The mode enables the concurrent mapping of Young’s modulus and the deformation and the topography of the sample [116]. Therefore, it can be used for investigating complex cellular and biomolecular structures to offer an in-depth quantitative multiparametric characterizations [117].

Viscoelastic mapping microscopy (VMM) is another mode that has its roots in research from multifrequency and bimodal AFM. This technique is a dynamic force-based mode that provides both imaging of the topography and maps of nanomechanical properties of soft-matter surfaces. In VMM, the cantilever is oscillated at two eigenmode frequencies. The first mode enables the recording of the surface features and loss tangent data whereas the second mode enables the recording of the frequency variations which can be used to obtain the stiffness of the sample. Thus, it is possible to obtain both the topography and map of the nanomechanical properties of soft-matter surfaces such as the contact stiffness and the modulus of elasticity [118]. Some advantages of this imaging mode are high spatial resolution, fast scanning, and low forces applied to the specimen.

Peak force tapping (PFT) was first introduced by Su and his co-workers [119] and is believed to possess advantages including the use of sinusoidal waves and subtraction of the background algorithm that allows the elimination of the parasitic deflection signal. With the use of PFT, it is possible to obtain the nanomechanical properties of samples at a faster scan speed with a very low minimum peak force in addition to high resolution mapping. The smaller force control is helpful in preventing any possible damage to the soft biological samples. Another benefit offered by the PFT is brought about by the use of tailor-made peak force microcantilevers with a longer tip length which allows a substantial distance between the cantilever and the sample, thus helping in the minimization of the hydrodynamic forces and background signal during operations [120]. Additionally, the PFT can also be employed in the study of biophysical properties by recording the single force–distance curve when the microcantilever probe is made to approach and retract from the sample surface. Thus, it is possible to characterize various mechanical properties, not necessarily limited to the adhesion force and dissipation energy.

The exhaustive list of AFM imaging modes is very long. Many of the recently developed modes are used for studying a number of biological samples, including proteins; small biological fibrils, like lipid membranes; amyloid fibrils; and viruses with the microcantilever as the sensing element [121,122]. Moreover, it has been demonstrated that malign and benign cell lines present significant differences in their viscoelastic response using MMM [123].

## 4. Methods of Cantilever Detection

Also of importance in the microcantilever systems are the readout methods that enable the determination of cantilever’s mechanical state at any specific time. This can be done with a good accuracy using either optical or electrical techniques. The optical methods that have been adopted in atomic force microscopy are typically laser-based and include optical lever techniques and laser interferometry [124]. These techniques can be used to detect a deflection of the microcantilever in the sub-nanometer regime. Other than optical readouts, electronic readouts comprising capacitance [125], piezoresistivity [126,127], piezoelectricity [128,129,130], and metal-oxide semiconductor field effect [131] have also been used for cantilever array detections; they show a good progress but are limited in performance by microfabrication complexity and lack biocompatibility.

### 4.1. Electron Tunneling Method

**Figure 5:**
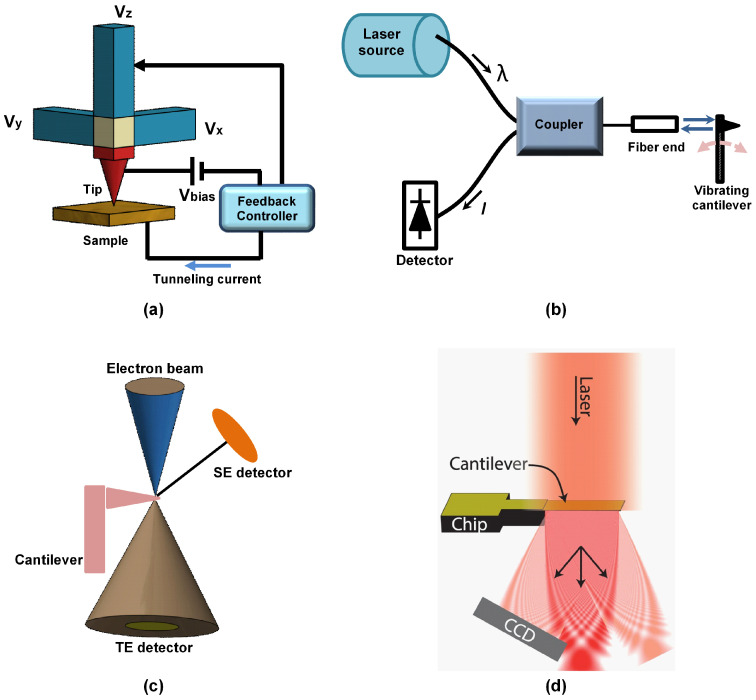
Schematics of (**a**) electron tunneling, (**b**) interferometric, (**c**) electron beam [132], and (**d**) optical diffraction microcantilever deflection detection systems [133].

In its infancy, the atomic force microscope microcantilever deflection was measured using the electron tunneling phenomenon. Here, the exponential dependence of the tunneling current between the scanning tunneling microscope (STM) tip and the cantilever on their separation distance is monitored. Typically, when a sharp, conducting tip is brought close to a conductive or a semi-conductive sample, electrons begin to tunnel from the sample to the tip or vice versa depending on the polarity of the bias voltage. The tunneling current varies with the tip–sample distance, and this variation in the tunneling current is the detector signal used to obtain the AFM images. This method offers a very high sensitivity but its main disadvantages are the reliance of electron tunneling on the surface conditions, difficulty in alignment especially in non-ambient conditions. Other limitations include the undesirable dependence of the tunneling detection on the effective spring constant, and changes in thermal drifts which greatly affect the force measurements. A schematic of the electron tunneling for measuring a cantilever deflection is shown in Figure 5a.

### 4.2. Interferometry Method

Interferometric displacement detection (see Figure 5b) is another method for measuring the displacement of a micro-cantilever [134,135,136] with sub-nanometer accuracy and with high resolution, but it is bulky and expensive. In fiber-optic interferometry, the optical interference in the micro-sized cavity between the cantilever and the properly cleaved edge of a single-mode optical fiber is used to detect small cantilever deflections. It is based on guiding the light entirely through an optical fiber and using a beam splitter to route light beams while the cleaved end and the reflective surface of the cantilever act as mirrors to produce the interfering patterns. The rationale behind this concept is that light is always delivered and collected through the same aperture that is several micrometers in diameter [137]. The high sensitivity and precision of a correctly calibrated displacement measurement makes optical interferometry a suitable method for measuring the small displacements of a cantilever. However, using optical fibers may induce additional imaging errors due to thermal drifts when the imaging duration is long in addition to special handling of the equipment to prevent stress during the positioning procedure [138].

### 4.3. Electron Beam Detection Method

The development of small cantilevers for high-speed AFM requires that the spot size of the laser beam directed to the back of the cantilever to be small (~1 μm). If not, the laser will spill-over to the sample surface causing problems with the detection of small cantilever deflections. Wagner et al. [132] proposed an electron beam, instead of laser beam, for detection of the small deflections of the cantilever. The electron beam is focused into a smaller spot size of few nanometers nearly 100 times smaller than the spot size of the laser in the optical lever scheme permitting the detection of the deflection of smaller AFM cantilevers with ease (see Figure 5c).

### 4.4. Optical Diffraction Grating

Optical diffraction grating has been implemented for detecting the deflection of the microcantilever as shown in Figure 5d. Here, the reflected laser light forms a diffraction pattern in which the intensity is proportional to the cantilever deflection in atomic force microscopy [133,139,140].

### 4.5. Piezoelectric Method

**Figure 6:**
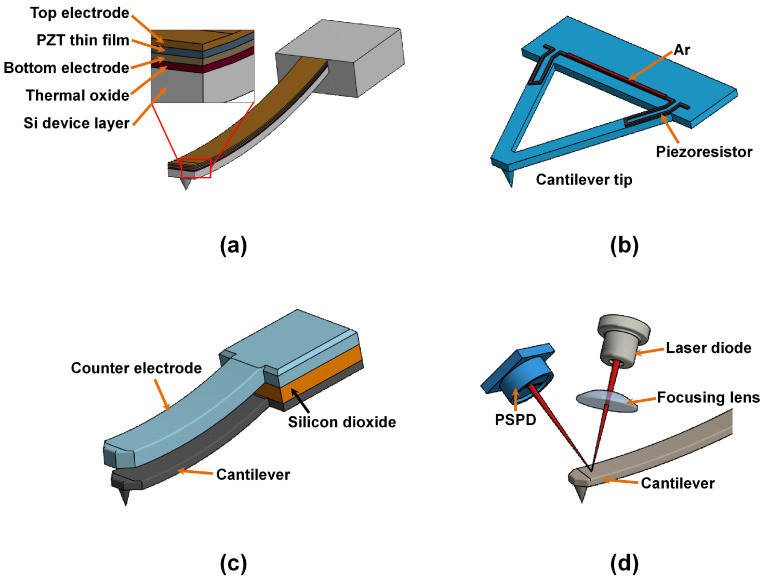
Schematics of (**a**) piezoelectric (adapted from [144]), (**b**) piezoresistive, (**c**) capacitive (adapted from [145]), and (**d**) optical lever microcantilever deflection detection systems [133].

In the piezoelectricity method, the electrical potential causes a mechanical stress on the microcantilever. The piezoelectric detectors have the advantages of consuming less power, easy to scale, possibility to be used in liquid environments, portability, and ability to withstand environmental damping. In addition, they can perform the dual function of actuation and sensing. Efficient actuation and elimination of optical interference from stray reflected light by the sample common in optical beam deflection method is a definite advantage. The on-chip actuation has the benefits of allowing multiple arrays of cantilevers on the same chip and permit feedback control at high frequencies [141]. However, thicker piezoelectric films are required for a significant output signals and also an electrical connection has to be made to the microcantilever. Recently, Moore and his co-workers [142] attempted to optimize the geometry of the piezoelectric microcantilever sensors to allow further miniaturization of such devices. They were able to achieve increased sensitivity and resonant frequency using optimized cantilever geometries compared to the conventional rectangular geometries. They formulated a means for utilizing the higher modes for the piezoelectric cantilevers by maximizing the microcantilever deflection and the measured piezoelectric charge response through strain partial distribution. Thus, they were able to increase the sensitivities of both the actuator and the sensor with a reduced sensor noise. Ruppert et al. [143] also demonstrated a method for optimizing the piezoelectric cantilever for multimode operations by altering the layout of the transducer depending on the strain mode shape without feed-through cancellation. A schematic typical of piezoelectric microcantilever deflection detection is shown in Figure 6a.

### 4.6. Piezoresistive Method

The pioneering work in the use of piezoresistors to sense the microcantilever deflection was proven by Tortonese et al. [146] from Stanford University in 1991. Numerous piezoresistive cantilevers have been developed since then by different researchers [147,148]. The idea is to position the p-doped thin resistors at high stress locations along the length of the beam [149]. Due to the piezoresistive effect, mechanical stress, induced within the resistors, leads to changes in their specific resistance. By biasing, via a fixed current, this change is converted into an electrical voltage signal (see Figure 6b). The stress sensitivity of the p-doped resistors linearly depends on the operating current. A typical example of a material that exhibits such characteristics is the doped single crystal silicon [150,151]. A deflection of the microcantilever induces stresses and therefore strains in the piezoresistor resulting into a change in resistance. Usually, these types of detectors are appropriate for an array of microcantilevers sensors and lab-on-chip devices. However, such sensors require sophisticated electronics to minimize parasitic effects and temperature drift as well as to maximize the signal-to-noise ratio. Other limitations making this method unpopular are poor sensitivity, thermal drifts and conductance, and thermal and electronic fluctuation noises [152].

A significant improvement in performance of such cantilevers with respect to piezoresistive deflection sensitivity and temperature stability has been achieved by using an integrated Wheatstone bridge configuration [149,153]. For example, Yu and his co-workers [154] used the 192 Wheatstone bridges to improve the sensitivity and noise levels of the piezoresistive microcantilevers made from single-crystal, microcrystalline, and amorphous silicon by varying the geometry, doping levels, and the annealing temperatures to achieve improved noise levels by up to 65%. Rasmussen et al. [155] also used a mathematical model to improve the sensitivity of a piezoresistive read-out system and was able to achieve a minimum detectable surface stress range [156].

The advantage of piezoresistive detection scheme compared to standard optical techniques is that neither additional optical components nor laser alignment are needed. Moreover, the read-out electronics can be integrated on the same chip using CMOS fabrication process [157]. The piezoresistive detection is unaffected by optical artifacts arising from the surrounding medium. The piezoresistive read-out can also be accomplished by an integrated gold resistor [158]. Xia et al. [159] have developed coated active scanning probes with piezoresistive deflection detection capable of imaging in opaque liquids devoid of the need of an optical system. The “Positive 20” polymer used for coating can withstand harsh chemical environments with high acidity (e.g., 35% sulfuric acid).

### 4.7. Capacitive Detection Scheme

The capacitive detection method involves the measurement of the capacitance between two electrodes. Usually the separation distance between the targeted electrodes influences the sensitivity owing to the inverse proportionality of the measured capacitance and the physical distance between the electrodes. The capacitive detectors mostly find wide application in gaseous media due to the sensitivity of the device to changes in the effective dielectric constant of the media between the two electrodes. However, this detection mechanism is not commonly used because of its many limitations [160]. Accurate measurement of the microcantilever deflection requires that the dielectric material between the electrodes remains constant throughout the experiment, although this is not always possible. Moreover, miniaturization of the capacitive cantilever has the limitation of lowering the overall sensitivity because of the direct proportionality of the capacitance and the electrode areas [161,162]. Some of the outstanding advantages of the capacitive detection system are high sensitivity, absolute displacement measurements and simple electronic design configurations [161]. A schematic representation of a capacitive detection scheme is shown in Figure 6c.

### 4.8. Optical Lever Method

Meyer and Amer [105] pioneered the optical beam deflection (OBD) technique in 1988, and it has proven to be a very reliable and simple method for detecting cantilever deflections [103]. Generally, the cantilever deflection is measured from the displacement of the reflected laser beam from the back of the cantilever with a quadrant photodiode. The reflected beam forms an optical lever system which amplifies small cantilever displacements. The movements of the beam are detected by using a position sensing photodiode, typically a quadrant photodiode. Sub-nanometer deflection sensitivity is routinely achievable using the OBD sensor. Compared to other displacement measurement methods, ease of implementation, ability to use a variety of cantilevers, ease of alignment, and low sensor noise levels make the OBD sensor the most adopted deflection sensor in commercially available AFMs. The schematic of a typical OBD sensor consisting of a laser source, a reflective cantilever, and a position sensing photodiode (PSPD) is shown in Figure 6d. In principle, as the free end of the cantilever bends, the position of the laser spot on the position sensing photodiode changes. Due to the fact that the distance between the cantilever and the detector is large, a small movement of the cantilever causes a significantly larger change in the laser spot position on the photodetector.

One of the problems that OBD presents to the user is the need for metal-coating the cantilever backside after fabrication to improve laser reflectivity. This procedure may induce unwanted deformation due to the bimetallic effect [132,138] but is helpful in some aspects as will be discussed later in the photothermal excitation section. Laser alignment of the three elements involved (cantilever, photosensitive photodiode, and the laser source) is also a tedious exercise for any new cantilever loaded in the AFM head [163].

## 5. Microcantilever Excitation Methods

Operating the microcantilever especially in the dynamic mode requires a clean resonance of the cantilever. Spurious resonances from the mechanical elements in the microscope are common problem especially when the piezo-acoustic method is used. The results are undesirable artifacts in the acquired images. Therefore, in order to improve the quality of the images for various AFM applications using microcantilever as the sensing element, the choice of cantilever excitation is important. In this section, the recent advances for exciting the cantilevers and strategies are discussed.

### 5.1. Magnetic Excitation

Magnetic excitation is one of the mechanisms used to drive AFM cantilevers and different approaches have been developed [164,165,166]. Basically, the object of this mechanism is to create a magnetic cantilever or probe that is driven outwardly by coil or solenoid. The early atomic force microscope cantilever probes were made from magnetic materials such as iron wires before silicon cantilevers became widespread, and this allowed a simple means of magnetic excitation. Attaching a magnet onto the surface of a cantilever using glue is one of the traditional methods of providing magnetic properties to a cantilever [166]. However, the additional mass caused by the magnet and the epoxy for mounting has the disadvantage of reducing the resonant frequency of the free microcantilever. Moreover, the difficulty of crushing the magnets to the desired size and mounting on the cantilever surface using epoxy was a problem. In order to overcome these limitations, the backside of the cantilever is usually coated with a very thin layer (between 0.03 and 0.04 μm thick) of a magnetic material such as cobalt using cathodic sputtering [167]. Despite the appealing nature of magnetic excitation resulting in clearer resonant peaks in liquid environments, it has several drawbacks [168]. Problems of reproducibility because of varying geometries of the magnet and its magnetic properties. The mechanical properties of the cantilever are altered as a result of the integrated magnet and the uniform repetitive magnetic cantilever production is not easy. Bending angle and stiffness are also altered by the coating. The sample may be contaminated by the magnetic metal ions. The process requires additional expensive equipment for deposition of the metal coating. Additionally, the electromagnet might cause local heating to the liquid cell. Lately, magnetostrictive actuation has been proposed where a change in the magnetic state results in a dimensional change of the magnetic material. For low frequency cantilevers typically less than 1 MHz, it has proven to be the most efficient method for multi-mode actuation especially in a liquid environment.

### 5.2. Brownian Motion

The collisions of liquid particles with the cantilever from Brownian motion can also excite the cantilever, thermally yielding a smooth cantilever response. However, the Brownian motion signal is hardly greater than the AFM sensor noise and therefore wrong measurements may be obtained [169,170].

### 5.3. Sample Excitation

Some researchers have also attempted to excite the sample rather than cantilever [171]. The existence of complicated dynamics and sub-harmonics makes this technique very difficult to achieve [172].

### 5.4. Electrostatic Actuation

**Figure 7:**
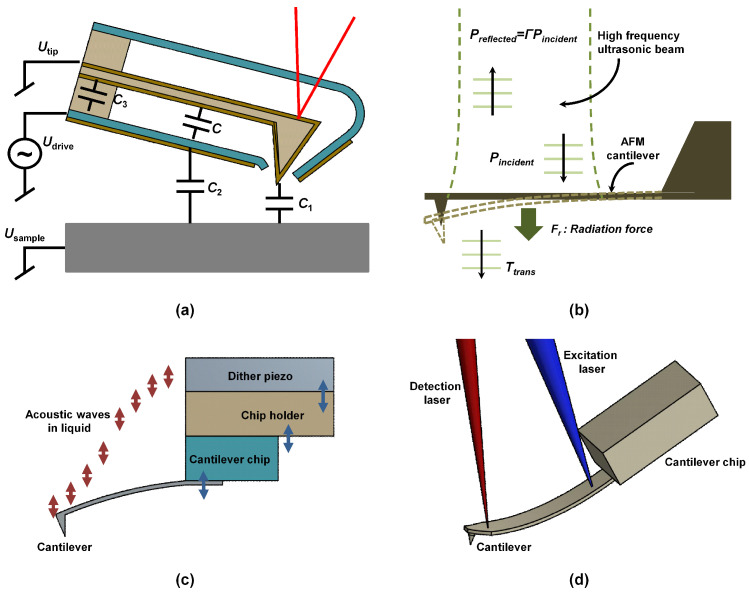
(**a**) A cross section showing the encased cantilever for electrostatic excitation. The capacitance $C_{1}$, $C_{2}$, and $C_{3}$ are parasitic capacitances whereas C is used for actuation [174], (**b**) acoustic radiation pressure excitation, (**c**) piezo-acoustic, and (**d**) photothermal excitation methods.

Electrostatic actuation is very versatile with the capabilities of actuating in both in-plane and out-of-plane directions. Here, the interaction forces between a conducting sample as well as a conducting cantilever probe are regulated by a bias voltage between the two [173]. One major limitation of electrostatic excitation method is the fact that both the cantilever and the sample need to be conductive. This condition greatly limits the sample that can be imaged as well as the materials that can be used for manufacturing the probe. Moreover, weak interaction forces require that a flexible cantilever must be used. Desbiolles et al. [174] demonstrated a method of exciting an encased cantilever using electrostatic technique (see Figure 7a) with a built-in electrode yielding smooth frequency resonance peaks both in air and liquid. The advantages of the built-in electrode drive and casing eliminates the need for any alignment, the use of only ac signal helps in reduction of the electrolytic production of gas bubbles, low noise, small cantilever amplitudes, thus reducing the tip–sample interaction forces and a reliable means to interpret the tip–sample interaction.

### 5.5. Acoustic Radiation Pressure Method

Some researchers have tried the acoustic radiation pressure method to excite the microcantilevers. One important merit of this technique is the ability to excite cantilevers of different materials and arbitrary shapes [175]. Basically, excitation is achieved by Langevin acoustic radiation pressure [176] which is created when a target cantilever is placed in the path of an acoustic wave beam at frequencies 100–300 MHz as shown in Figure 7b. When this pressure is focused at the focal plane of the lens, localized forces are generated to excite as well as evaluate the dynamic and static characteristics of the cantilever.

### 5.6. Piezo-Acoustic Excitation

In piezo-acoustic excitation, a small piezoelectric actuator is positioned close to the cantilever to indirectly excite the cantilevers. This method is by far the most common in AFMs. This is partly because of ease of implementation, ease of operation, and cost effectiveness. Usually, several parts are involved in the excitation process because the piezo-actuator cannot be mounted directly on to the cantilever. Therefore, the excitation begins from the cantilever holder to the cantilever via the chip on which the cantilever is mounted. Although this technique works relatively good in both air and vacuum environment, this in-direct excitation of the cantilever results in mixed resonances due to mechanical impedances of the piezo, cantilever holder, and cantilever base. This leads to the so called “forest of peaks” in liquid environment where the quality factor is low [177]. The unwanted mechanical resonances may not only affect the detection laser in the optical beam bounced technique but also makes it extremely difficult to choose the correct resonant frequency of the cantilever [178] because of the complex mechanical coupling. Moreover, it has been noted that piezo acoustic excitation may cause sample transience and movement of the molecular sample due to sonication [165]. Attempts to minimize the forest of peaks in low Q-factor environments by designing special cantilever holders have been fair at best [179,180]. Another method that have been proposed to help minimize the forest of peaks is by integrating a piezoelectric material such as zinc oxide (ZnO) on the cantilever [181]. The piezoelectric material provides a means of exciting the cantilevers at fast speed (greater 10 kHz) in tapping mode. The ZnO actuator can have dual function of exciting the cantilever and providing motion in the Z-direction for the tip–sample distance regulation. It is always desired that the measured quantity should be the variation in tip movement alone. However, this is not the absolute case in piezo-acoustic excitation. This is due to cantilever bending which is not exactly equivalent of the tip motion [168]. A schematic of a typical piezo-acoustic cantilever excitation method is shown in Figure 7c.

### 5.7. Photothermal Excitation

The photothermal excitation method is based on the fact that the microcantilevers can be easily modified by coating the upper surface with a thin layer of a different material. Owing to their difference in the coefficient of thermal expansion, when the composite material is subjected to a temperature change, the microcantilever deflects. In atomic force microscopy, power modulation of a focused laser beam at the back of the microcantilever at a designated drive frequency [182] or joule heating [183] are the two major methods used to achieve the desired heating. Photothermal excitation method has a few drawbacks including low displacement and low efficiency [184] and the difficulty of exciting the higher modes. The advantages of photothermal excitation are however enormous when compared to other conventional; high bandwidth [185,186], sharp resonant peak in liquid and ease of implementation as shown in Figure 7d and the ability to use as fabricated microcantilevers without coating. One problem with the bimorph microcantilevers is the fact that longitudinal thermal diffusion inhibits the lateral bending in diffusion direction. However, the implementation of the photothermal excitation on single crystal by Nishida and his co-workers [186] a decade ago was a major breakthrough in solving this problem. Thus it is possible to precisely excite microcantilever modes of higher frequencies. Another common method of exciting different modes in single crystal microcantilevers is through varying the position at which the focused laser spot hits the back of the cantilever.

### 5.8. Optical Excitation

**Figure 8:**
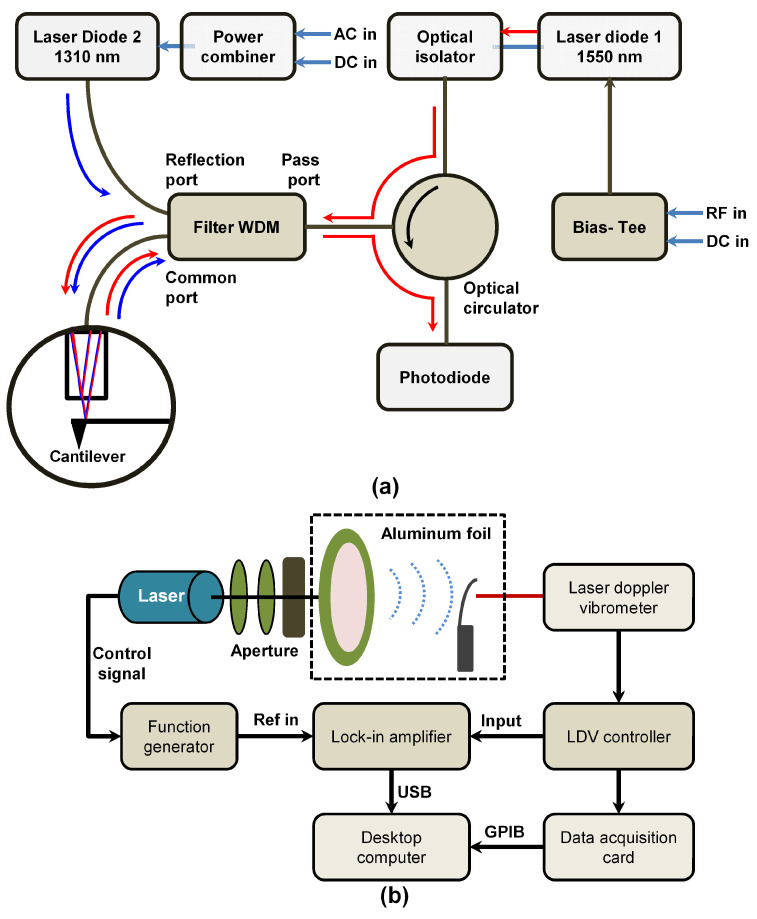
(**a**) A schematic of the optical excitation method for the microcantilever proposed by Miyahara et al. [187]. (**b**) Schematic of the experimental set-up for the laser induced photoacoustic excitation method for the microcantilever [188].

Miyahara and his co-workers [187] have proposed a new method for exciting a microcantilever sensor by combining two laser in a single-mode optical fiber using a filter wavelength division multiplexer (FWDM) to achieve both excitation and detection. With the set-up it was possible to eliminate the spurious mechanical resonances associated with the piezo-acoustic excitation method (see Figure 8a). The interference of the returning light from the back of the cantilever and the fiber end goes back to the FWDM that helps to block the reflected excitation laser signal and only allows the detection laser to pass to the photodetector through an optical circulator. Modulation was achieved by modulating the drive current with a power combiner. This method allows an easy modification to the existing AFMs that use the fiber-optic interferometers for detecting the microcantilevers.

### 5.9. Laser Induced Photoacoustic Excitation

Remote excitations of microcantilever based sensors by laser-induced photoacoustic (PA) waves have recently been reported by Gao et al. [188]. This excitation technique shown in Figure 8b typically relies on the generation of PA waves from an optical absorber, followed by effective delivery of these propagating PA waves on the lever surface through a medium. It may enable microcantilevers to be used as photoacoustic sensors and presents itself as a substitute method for detecting small signals by eliminating the heating effect common in other optical excitation methods. However, these potential applications call for a comprehensive understanding of the microcantilever response to the laser-induced PA waves.

## 6. Fabrication, Modification, and Functionalization of AFM Microcantilevers

### 6.1. Fabrication

Cheap, miniature, and reproducible fabrication of microcantilevers has been possible from silicon, silicon nitride, silicon oxide, or silicon-on-insulator (SOI) by taking an advantage of the batch silicon micromachining techniques developed for integrated circuits (IC) and CMOS process technologies [189,190,191,192]. In fact, wide applications of microcantilevers in industries and most research facilities have been made possible by the fact that they can be mass-produced and they are easy to be miniaturized. The microcantilevers are available in various dimensions, shapes, and sensitivity. Often, the geometry of the microcantilever is dictated by the mode of detection. The dimensions of the microcantilevers range from 100 to 500 microns in length and below 5 micrometers in thickness. Typical shapes are the “T” (rectangular) or the “V” (triangular) with a sharp tip mounted on the free end. In the recent past, the need for small high bandwidth cantilevers has risen for high-speed atomic force microscopy, and similar technologies have been used for their production [193,194,195]. Many investigators with a full access to well-established micromachining facilities have delved in the fabrication of microcantilevers.

Other researchers have attempted the fabrication of microcantilevers using organic-based materials such as SU-8 and Polydimethylsiloxane (PDMS) [53,196] because of their low modulus of elasticity and versatile and simple processing procedures. By using the bottom-up approaches, the microfabrication process of the SU-8 microcantilevers has a high output. Using polymer cantilevers has been shown to outperform the silicon or silicon nitride microcantilevers particularly concerning the imaging speed of the atomic force microscopy (AFM) in tapping mode by up to one order of magnitude. However, the polymer microcantilever tips do not often have the required sharpness and durability for imaging in contact or contact resonance mode [197]. Therefore, a way to combine the high imaging bandwidth of polymer cantilevers with the sharp and wear-resistant tips is necessary for a future adoption of polymer cantilevers in routine AFM uses [198]. An attempt by Martin-Olmos and co-workers [199] to coat SU-8 microcantilever and the tips with wear-resistant graphene was unsuccessful in creating sharp tips.

The SU-8 polymer microcantilevers have been applied in the study of different biological phenomenon. High-resolution AFM images of DNA plasmid molecules have been presented by Genotel and co-workers [200]. Additionally, the polymeric SU-8 microcantilevers have been applied in high speed amplitude modulation AFM and shown improved performance due to their high mechanical bandwidth and low mechanical quality factor (Q-factor) [201]. In a recent article, Kramer et al. [202] proposed a simple method of fabricating ready-to-use micro-fluidic microcantilevers by using a combination of two-photon polymerization and stereolithography 3D additive manufacturing processes. The method offers an inexpensive, fast and more flexible way of fabricating the microcantilevers. A microcantilever of dimensions 564 μm long, 30 μm wide, 30 μm thick was fabricated with a spring constant of about 0.0037 N/mm. The reported micro-fluidic microcantilevers were used to puncture the cell membrane and aspiration of a single cell.

### 6.2. Microcantilever Tip Fabrication

A majority of the imaging and surface characterization done using an atomic force microscope are carried out with microcantilever probes as the sensing elements. They form part of the consumable items required for the running of the AFM especially when high spatial resolution imaging is needed. Several methods have been used to create the sharp probes on the microcantilever suitable for high resolution imaging. Zenhausern et al. [203] used scanning electron microscope (SEM) to fabricate sharp carbon tips at the end of commercial silicon nitride cantilevers through electron beam induced deposition (EBID) technique. Akiyama and his co-worker reported a successful fabrication of a sharp tip with a radius of curvature of less than 5 nm in a microcantilever using the focused ion-beam (FIB) method [204]. Tay and Thong used a simple field emission induced growth (FEIG) of a tungsten nanowire enabling the production of sharp and robust high-aspect ratio microcantilever probes for AFM applications. They were able to achieve probe lengths up to 1500 nm with a tip radius of less than 2 nm [205]. Dremov and his co-workers demonstrated the fabrication of robust, conductive microcantilever tips suitable for scanning contrast or Kelvin probe force microscopy using a single multiwalled carbon nanotube (MWCNT) by employing dielectrophoresis technique from the MWCNT suspension [206].

**Figure 9:**
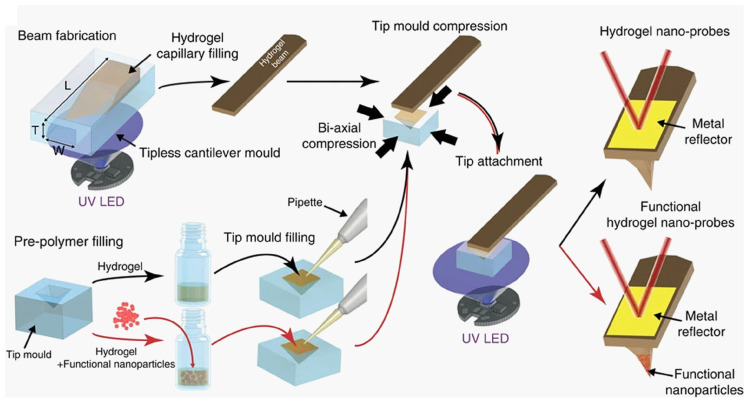
Fabrication method for hydrogel AFM probes. A tipless hydrogel cantilever is first prepared by ultraviolet curing of the pre-polymer solution introduced into the cantilever beam mold. The tipless hydrogel cantilever then makes contact with a tip mold filled with pre-polymer solution with or without encapsulated functional elements, followed by a second round of ultraviolet exposure to cure the hydrogel in the tip mold. This results in the firm attachment between the cantilever and tip. Before the second ultraviolet exposure, the hydrogel-filled tip mold can be optionally deformed by applying bi-axial compressive strains to facilitate tunable tip sharpness and aspect ratio. Reprinted from the work in [207].

Lee and his co-workers [207] demonstrated a process for fabrication of photopolymerizable hydrogel nanoprobes with tunable mechanical properties, allowing an easy encapsulation of nanomaterial with differing sizes and different possibilities of functionalization [208,209,210]. Additionally, the hydrogel material on account of its softness could provide a good microcantilever for biological and soft matter AFM applications [211]. The hydrogel-based cantilevers are found to have widely tunable and low mechanical stiffness suitable for sensitive nanomechanical measurements of soft matter. The multifunctional and programmable capabilities of the hydrogel nanoprobes were also demonstrated including temperature sensing, material delivery, and local heating. The process involves using ultraviolet light-induced curing of a pre-polymer solution introduced into a mold in order to fabricate the tipless hydrogel cantilever. The tipless microcantilever is then brought into contact with a tip mold filled with a pre-polymer solution. Curing is achieved by exposure of the hydrogel in the tip mold using a secondary ultraviolet resulting in a strong connection between the tip and the cantilever before coating to increase reflectivity [207]. The hydrogel filled tip mold can be optionally deformed to apply a compressive strain to enable tunable tip sharpness and high aspect ratio. A summary of the fabrication method for the hydrogel AFM micro-cantilever is shown in Figure 9.

### 6.3. AFM Microcantilever Modification

As-fabricated microcantilevers work effectively in many AFM applications. In fact, it has been demonstrated that when they are operated in the dynamic flexural mode, they exhibit relatively good sensitivities [212,213,214]. The principle of operation of such microcantilevers is based on a shift in the resonant frequencies owing to the fluid moved by the microcantilevers during vibration. For density sensor applications, a decrease in mass density of the fluid surrounding the microcantilever causes the equivalent effective mass of the microcantilever to decrease, thereby causing the resonant frequency to decrease or vice versa [215]. The advantages of the uncoated microcantilevers are numerous such as reductions in aging effects, thermal drift and longtime response [216]. The thermal drift is a result of increased heat from the surface due to a temperature gradient.

The uncoated microcantilevers offer low sensitivity and are non-selective when used in special sensor applications for gas detection or density measurement. However, the uncoated microcantilevers suffer from a low level of reflected laser power from the back of the cantilever. In the application of the microcantilevers for photothermal excitation, the most common principle used is the bimorph that requires the surface of the microcantilever to be coated with a secondary material having a different coefficient of linear expansion. The reflective metal coating with a thickness of a few tens of nanometers offers a benefit of amplifying the reflected laser beam off the microcantilever surface thereby enhancing the signal-to-noise ratio. In addition, the coated microcantilever can prevent the interference between the reflected beam from a very reflective sample. The main coating materials used in microcantilevers are gold, platinum, and aluminum. Although the use of aluminum for surface coating is cheap and provides good reflectivity, it is not suitable for use in most biological buffers or solvents because it is highly unstable or even dissolvable in a liquid environment. The use of gold on the other hand offers stability because it is biologically and chemically inert. Platinum is often used for electrical or magnetic measurements. Recently, Xia et al. [159] tried different polymer materials such as M-Bond 610, 2K-Epoxy, M-Bond 43B, and Positiv 20 for active AFM microcantilevers using dip coating process. Positiv 20 polymer gave superior outcome in terms of coating layer thickness, good bond capability and less corrosiveness to chemical attack. The developed polymer coated active cantilevers allowed imaging in opaque liquid environments such as crude oil, vinegar, and immersion test in blood sample.

### 6.4. AFM Probe Functionalization

The atomic force microscope microcantilever tips have the flexibility of being functionalized for chemical and biological applications to allow the attachment of the sensing molecules. Prior to functionalization, sometimes the cantilevers are gold coated to provide a convenient platform for chemical or biological functionalization by taking advantage of thiol-gold chemistry [217]. The customization possibilities for tips are endless. With the functionalized tips, the AFM is capable of providing sensitive tool for measuring and mapping surface chemistry and quantifying repulsive and adhesion forces related to the biological samples and inorganic materials. This is made possible by controlling the chemical interactions between the AFM tip and the sample. Functionalization typically involves chemical modifications of the tips using particular functional groups in order to carry out a specific function in the system. Before functionalization, the tips should be carefully inspected for quality in terms of the material, tip radius, shape and size, resonant frequency, and spring constant. When a low quality tips are used, it can lead to imaging artifacts. The silicon nitride cantilevers are preferred for studies involving molecular recognition [218]. Their biggest advantage is the commercial availability of several different silica precursors highly suitable for decorating the AFM tips with the desired functional groups.

A number of techniques have been proposed to functionalize the AFM microcantilevers for use as chemical or biological sensors. For example, Daza et al. [219] attempted the functionalization of a reliable and robust AFM microcantilever tips by using the activated vapor silanization (AVS) process. The functionalized tips were able to withstand repetitive interactions with a model graphite substrate under relatively harsh conditions with no damages to the tip. The process involved pre-heating the tip to create a high density of hydroxyl (-OH) groups on the surface. The hydroxyl (-OH) groups may then react with an organosilicon compound such as aminopropyltrietoxisilane (APTES) terminated in a reactive group such as amine. More sophisticated functionalization methods have also been proposed and explored, such as plasma enhanced chemical deposition (PECVD). The use of PECVD-functionalization, however, requires an activation step of the substrate, which can be performed by creating oxygen-containing plasma before starting the functionalization process [220]. This activation step is supposed to create a high density of hydroxyl groups on the surface to which the APTES molecules may bind covalently. Even though PECVD-functionalization allows deposition of thick layers, the thickness of the functionalized thin film tends to be restricted to a few nanometers (5–10 nm) [219].

Other processes that have been used to functionalize AFM microcantilever tips include self-assembled monolayer (SAM), vacuum thermal evaporation, and sputtering. The self-assembled monolayer functionalization method involves dropping specific reagents on the tip of the microcantilevers and rinsing after a time duration with a different reagent. This is followed by immersion into a solution or ultra-pure water. During the process, functional groups such as –CH${}_{3}$, –COOH, or organosiloxane monolayers are formed on the tip under the controlled conditions on a gold coated surface. By using the self-assembled monolayers to functionalize the AFM tips, a window of opportunities have been opened that enables understanding diverse interfacial phenomena, self-organization, and structure–property relationship [221,222,223]. Sputtering on the other hand is used to functionalize the microcantilever AFM tips to induce specific properties such as ferroelectricity, thermal, and electrical conduction and optical reflectivity [224].

Operation of the atomic force microscope in the colloidal probe mode has also proven to be effective in quantitatively measuring the nanoscale interactions at biopolymer interfaces, drainage of thin films, lubrication theory, mechanical properties of cells and deformation of colloidal droplets [225,226,227,228]. It involves attaching a colloid sphere below the microcantilever thus allowing the measurement of the surface phenomena with sub-nanometer and pico-newton resolution.

The functionalized AFM tips using various binding groups have been used widely in the past to study interfacial interactions. For example, Ma and his co-workers [229] investigated the generated adhesive force between a hydrophobic microcantilever tip and immobilized oligopeptides surface. It is possible to quantify and identify the receptor–ligand interactions usually in the range below 100 pN [230]. Different force spectroscopy techniques such as optical tweezers, atomic force microscopes, and biomembrane force probe have been used to obtain quantitative information about the adhesion force below nN range [231]. Often, the optical tweezers and the biomembrane force probes methods are less preferred because they are limited in the detachable adhesion force range [232]. For atomic force microscope techniques, the single-cell force spectroscopy mode is employed to study the cell to cell interactions mostly carried out in their physiological buffer solutions and conditions. Additionally, it has a significantly large range of detachable forces up to 1 μN in addition to the precise temporal and spatial control over the experiments. For example, Zhang and co-workers [233] used a soft microcantilever functionalized with the cancer cell using biotin-conA brought in contact with the endothelial cell monolayer grown on a surface allowing the detection global adhesion strength and breakup of receptor–ligand bonds.

## 7. High-Speed Imaging

In spite of many positive aspects, one of the most limiting disadvantages of typical atomic force microscopes is the slow scanning speed. For most commercial atomic force microscopes, image acquisition takes several to tens of minutes [234] since the line scan speed is typically around 1 Hz. Fortunately, there have been many improvements in the imaging speeds of AFM, especially in the past decade. Several technological hurdles should be overcome to improve imaging speeds, and these include the slow data acquisition systems [235,236], low resonant frequency of the nano-positioners and scanners [234,237,238], low bandwidth of the feedback controller [234,237], and low resonant frequency of the microcantilevers [235,236,239,240]. An effective means to excite the microcantilevers in the MHz regime is also needed.

Extending the speed capabilities of AFM has inspired many researchers to do an extensive work in this area. Significant efforts have been put on developing high bandwidth scanners and cantilevers [235,241], high bandwidth cantilever deflection detection systems [242,243,244,245], and fast and robust feedback system with Z-scanner [109,246]. The heart of atomic force microscope is the microcantilever sensor that interacts with the sample to measure the desired surface features. Interestingly, the microcantilever was the biggest obstacle for raising the speed of the AFM due to the limited bandwidth of typically available cantilevers. High bandwidth of the microcantilevers was achieved by the advent of the robust, commercially available ultra-small cantilevers that enabled the reduction of the overall microcantilever mass [181,237,247]. Typical dimensions of the ultra-short microcantilevers are a few microns in length, about 10 times smaller than conventional cantilevers, a resonance frequency above 1 MHz and a low force constant typically in a few nN/m [248] compared to the conventional tapping mode cantilevers. A summary of the properties of the commonly available ultra-short cantilevers (AC10 and AC7) and regular cantilevers (MLCT-E and AC40) are compared in Table 2. The miniature cantilevers also have low spring constant (k), reduced coefficient of viscous drag (β), and low quality factor (Q). The low quality factors and high resonance frequencies are required for the ultra-short cantilevers to have a small response time. The total thermal noise $\sqrt{\frac{k_{B}T}{k_{c}}}$, where $k_{B}$ is Boltzmann’s constant, $k_{c}$ is the spring constant, and *T* is the temperature in Kelvin, is distributed over frequencies up to slightly above the resonance frequency, $f_{c}$. Thus, a cantilever with a higher $f_{c}$ has a lower noise density.

AC10 and AC7 are the commonly used probes because they have silicon nitride tips that allow easy functionalization compared to the carbon AFM tips deposited using the electron beam method [3]. The ultra-short cantilevers require a small laser spot size for the detection of the cantilever deflection. The small laser spot required for the ultra-short cantilevers are provided for either by using the power micro-lenses or using a microscope objective [235,237].

The development of high-speed atomic force microscopy (HSAFM) has enabled the generation of AFM images at video rate and recording of force–distance curves at high speeds [237,249,250,251]. The introduction of AFM to capture the live actions of biomolecules at high spatial and temporal resolutions has been demonstrated by HSAFM [252,253]. AFM-based recognition imaging and force spectroscopy allow unbinding force mapping of receptor–ligand interaction sites on a lipid membrane at the single molecule level [254].

HSAFM is also a force spectroscopy tool. In force spectroscopy, the force–distance curves are obtained. Typically, there are different force spectroscopy approaches based on the experimental setup such as the functionalization of the tip or the type of distance modulation used. Single-cell force spectroscopy and single-molecule force spectroscopy are used in the study of biomolecular or cell adhesion processes at the single-biomolecule level [117,255,256]. Peak force tapping and force volume methods are the other two force spectroscopy methods applied in the study of the nanomechanical response of polymers, cells, inorganic, and organic interfaces [257].

High speed capabilities have been useful in the study of time-dependent dynamic and kinetic processes that involve melting, crystallization, growth, and annealing of several surfaces including polymers, crystals, and biological molecules [3]. The high-speed AFMs provide a way for understanding the mechanical properties of biological systems and processes at the nanoscale [252,258]. In fact, many biological processes present in many organisms occur over a short time scale. It is possible to visualize cellular dynamics and various proteins at video rates [249,259]. Kodera and his co-workers demonstrated a real-time observation of walking myosin V on an actin filament [250]. Yu et al. [260] applied the high-speed atomic force microscope with ultra-short cantilevers to unfold the individual bacteriorhodopsin molecules in a native lipid bilayer. Matusovsky and co-workers [261] studied the 3-state model of activation of cardiac thin filaments isolated as a complex and deposited on a mica-supported lipid bilayer. They realized that the successful imaging of the regulatory proteins tropomyosin and troponin complexes is dependent on the force applied by the cantilever tip because of their low affinity to F-actin. Thus, a small force should be applied neither to break the electrostatic bonds within the regulatory units of the cardiac thin filaments nor reconstituted F-actin–tropomyosin–troponin complex.

## 8. Microcantilever Sensors in AFM Applications

The applications of AFM microcantilevers are enormous, ranging from solving problems in different areas such as energy, health care, and agriculture, to handling environmental and process industrial issues. For example, nano-biosensors have been used to monitor the treatment procedures and detection of contaminants and heavy metals in industrial processes [91,262]. The microcantilever nano-biosensors are easy to use, sensitive, small, fast, and versatile in terms of detection and monitoring [263]. Some of the limitations of nano-biosensors, however, include the possibility of multi-agent detections by the conversion of bimolecular activity into a measurable quantity and disturbance from the fluid medium during the measuring and temperature control. Typically, the microcantilever deflection or the frequency shift due to the mass change is used to determine the concentration of the target parameters [264].

Rigo et al. [265] developed an efficient, highly sensitive nano-biosensor by functionalizing a microcantilever with urease enzyme, and they were able to detect heavy metals such as cobalt, zinc, nickel, and lead in water. The nano-biosensor was able to achieve a detection limit of parts per billion for the 30 days of storage showing a relatively good stability. The functionalization process was performed on the upper surface of a gold coated silicon cantilever using self-assembled monolayers (SAM) process, by cross-linking agents 1-ethyl-3-(3-dimethylaminopropyl) carbodiimide (EDC), and N-hydroxysuccinimide (NHS). The heavy metals present in the water solution bind to the active site groups of the urease enzyme by reacting with the sulfhydryl groups. The reaction causes a stress tension on the cantilever surface, resulting in a deflection measured by the voltage change of the cantilever nano-biosensor.

Muenchen et al. [266] functionalized a microcantilever for use as a biosensor using peroxidase from vegetables for the detection of glyphosopahe herbicide with a wide spectral range. The deposition of the peroxidase enzyme on the cantilever was done using the self-assembled monolayers (SAM). The adsorption of the glyphosate resulted in a change in the surface tension causing a conformal change in the structure of the peroxidase enzyme.

Rezaee et al. [267] presented a numerical model of an electrically actuated biosensor for identification and characterization of different bio-particles. The process involved coating the microcantilever with receptor chemicals followed by biasing before analyzing the pull-in instability characteristics.

Sutter et al. [268] combined high-speed atomic force microscopy and X-ray crystallography to study the structure and dynamics of the bacteria micro-compartments shell facet assembly at the molecular resolution. Diverse insights into the structure revealed the formation of single layer sheets of a uniform orientation from pre-assembled shell hexamers. The hexamers could also dissociate and combine into an assembled sheet showing the flexibility in the intermolecular interaction. Having a better concept of the bacteria micro-compartments help researchers understand their control and potential use in nanoreactors and molecular scaffolds.

Possas-Abreu et al. [269] recently detected the binding of 2-isobutyl-3-methoxypyrazine to the immobilized odorant binding proteins (OBP) using a grafted OBP on a diamond micro-cantilever by applying MEMS technology. From their work, an approximated 108 molecules of 2-isobutyl-3-methoxypyrazine was bound to the immobilized OBPs showing the possibility of using them as reliable vapor biosensors.

Improvement in the binding efficiency of microcantilever array biosensor has been demonstrated by Liu et al. [270] using the Yersinia detection method. They introduced an antibody to increase the capture efficiency by enhancing the binding sites and reaction efficiency.

Bertke et al. [271] developed a sensitive micro-cantilever based particulate matter detector with a combined electrostatic on-chip ultra-fine particle collection and separation. The microcantilevers had collection electrodes in order to attract the charged particles naturally and an integrated microchannnel to enhance the efficacy of the particle collection. The detection limit for the miniature sensors is about 1 μg/$m^{-3}$.

Guillaume-Gentil et al. [272] presented a simple method for extracting the endogenous soluble elements from single cells using fluidic force microscopy for further analysis. The process involves the insertion of the microcantilever tip with a triangular aperture of about 400 nm on the front side of the pyramidal tip inside the single cells. After which the extracted fluid fills the probe with the help of a negative pressure. Quantification of the extracted endogenous elements was made possible by using an integrated optical microscopy. Because of the gentle and controlled force offered by the flexible microcantilever during the operation of fluid force microscopy, it was observed that even after the extraction of large volumes of cytoplasm molecules, it was possible for the cells to undergo cell divisions and stay alive. The method demonstrates that there is a potential of extracting smidgen elements for molecular analyses. In addition, it is possible to use undiluted samples for third generation sequencing technologies, building and analysis of the artificial cells and determination of epigenetic changes.

Microcantilever-based biosensors arrays have become to be reliable and very precise instruments for the detection of cancer diseases. Wang et al. [273] reported antibody functionalized microcantilever arrays for the detection of liver cancer. They reduced the adsorption-induced variation of the cantilever stiffness by making a micro-cavity at the end of the microcantilever for local immobilization of the antibody. In addition to the analytical model, they were able to increase the detection sensitivity of the mass of the detected antigen and the overall accuracy of the liver cancer biomarker detection.

In another article, Kamble et al. [274] reported the detection biomarker for early diagnosis of diabetes using piezoresistive microcantilevers and inter-digitated electrodes. The principle is based on the high sensitivity and selectivity of tungsten trioxide towards acetone in an environment filled with selected volatile organic compounds. Screen printing was used to deposit the tungsten trioxide on the inter-digitated electrode fingers, and the resistance measurement was done by using an electrometer. This piezoresistive-based microcantilever work showed the high sensitivity of 2.1 towards 10 ppm acetone at 250 ${}^{\circ }$C.

Recently, Kim et al. [275] reported a universal means of measuring the binding affinities of nivolumab antibody drug towards the target. The method involved coating the surface of a tipless microcantilever with nanocapsules followed by the immobilization of the nivolumab molecules through binding between the antibody and the target protein. The nivolumab-coated AFM cantilever and the T lymphocytes on which programmed cell death 1 molecules expressed are used for investigations. In the experiment, the rupture forces between the programmed cell death 1 molecules and the nivolumab molecules on the microcantilever were monitored. It was demonstrated that this method could allow a comparison of the affinities of different antibody drugs towards a single cell because it does not involve a chemical treatment.

Korayem et al. [276] recently used the microcantilever-based atomic force microscope to obtain various mechanical and physical properties of the head and neck cancer cells. These properties include the modulus of elasticity, cell topography, and viscoelastic properties. From the measurements, the average adhesion force recorded in contact mode for a cantilevers operated in air was 2.47 nN. The research is a step ahead towards characterizing head and neck cancer cells in a heterogeneous population.

## 9. Conclusions and Prospects

This review article presents the recent developments in the microcantilevers and their applications in various fields. It has been shown that microcantilevers play a pivotal role in the detection of various phenomena using atomic force microscope. A variety of methods for detecting the deflection of the microcantilever have been discussed and the improvements in the recent years have been done to accommodate the ultra-short microcantilevers. It is possible to fabricate microcantilevers both from silicon and selected polymers sensitive to bending moments owing to their lower spring constants. The high sensitivities of microcantilevers have made it possible to investigate complex and advanced chemical and biological problems. Different methods for coating and functionalization of the microcantilever surface for chemical and biological purposes have been assessed.

There is a constant progress in the microcantilever applications with novel detection strategies being developed for higher sensitivities in the atto-newton regime and easier operations. Latest applications of microcantilever-based chemical and biological sensors have been presented. The sensors are reproducible, cost effective for fabrication, robust, easy to handle, power efficient, and small.

The developments achieved in the last few years in both hardware and software for the atomic force microscope has enabled imaging at unprecedented speeds. Additionally, the measurements of the mechanical properties and other surface phenomena in air, aqueous media and at cryogenic conditions have also been conducted with relative ease. Biomaterials and soft matters that seemed impossible to image in the past is now possible by the development of the ultra-fast, flexible microcantilevers.

It is evident that the microcantilever-based sensor is still a work in progress allowing researchers to explore more areas of applications. Further research is required for the development and realization of more robust microcantilever systems for the future applications.

## Figures and Tables

**Table 1:** Areas of applications for the microcantilevers.

S/No	Areas of Application	Examples
1	Biomedical applications [86,87]	Biosensors (DNA, antibodies
		proteins, viruses, and microorganisms)
		Diagnostics
		pH sensors
2	High frequency resonators [88,89,90]	Chemical sensors
3	Food production and safety [91]	Detection of heavy metals in water
		To detect concentrations of herbicides
		Changes in pH
4	RF switching [92,93,94]	Broadband switches
		Switches for wireless communication
5	Atomic force microscopy [3,95,96]	Live cells
		Reaction processes of DNA
		Biomolecules
6	Environmental monitoring [97]	Temperature detection
		Humidity detection
		Heat changes
7	Read and write storage devices [98]	Storage devices
8	Home land security [99]	Detection of terrorism weapons
		Explosives detection
		Monitor missile storage and maintenance needs
9	Energy [100,101]	Energy harvesters

**Table 2:** The comparison of the properties of commonly available regular (MLCT-E and AC40) and ultra-short (AC10 and AC7) microcantilevers.

Property	MLCT-E	AC40	AC10	AC7
Shape	V-shaped	Rectangle	Rectangle	Rectangle
Length (μm)	140	38	8	6
Width (μm)	18	16	2	2
Thickness (nm)	600	180	130	130
K (pN/nm)	112	102	87	592
$f_{0}$ in liquid (kHz)	7	31	431	1231
Q-factor in liquid	1.7	1.6	0.8	0.7
β (pNs/μm)	4.59	0.82	0.03	0.05

## Abbreviations

The following abbreviations are used in this manuscript.

ACM	Aperture Correction Microscopy
AFM	Atomic Force Microscope
APTES	Aminopropyltrietoxisilane
CM	Contact Mode
CMI	Contact Resonance Imaging
CMOS	Complementary Metal Oxide Semiconductor
EBID	Electron Beam Induced Deposition
EDC	1-ethyl-3-(3-dimethylaminopropyl) carbodiimide
FEIG	Field Emission Induced Growth
FIB	Focused Ion-Beam
FLIM	Fluorescence Lifetime Imaging Microscopy
FMM	Friction Mode Microscopy
FRET	Förster Resonance Energy Transfer
FWDM	Filter Wavelength Division Multiplexer
HSAFM	High-Speed Atomic Force Microscopy
IC	Integrated Circuits
KPFM	Kelvin Probe Force Microscopy
LSCM	Laser Scanning Confocal Microscopy
MEMS	Microelectromechanical Systems
MFFM	Multifrequency Force Microscopy
MFM	Magnetic Force Microscopy
MMM	Mechanical Mapping Mode
MWCNT	Multiwalled Carbon Nanotube
NCM	Non-Contact Mode
NHS	N-Hydroxysuccinimide
OBD	Optical Beam Deflection
OBP	Odorant Binding Proteins
OCLSM	Optical Confocal Laser Scanning Microscopy
OFM	Optical Fluorescent Microscopy
PDMS	Polydimethylsiloxane
PECVD	Plasma Enhanced Chemical Vapor Deposition
PI	Phase Imaging
TERS	Tip-Enhanced Raman Spectroscopy
SAM	Self-Assembly Monolayer
SEM	Scanning Electron Microscopy
SOI	Silicon-on-Insulator
SNOM	Scanning Near-Field Optical Microscopy
SRFM	Super-Resolution Fluorescence Microscopy
STEDM	Correlative Stimulated Emission Depletion Microscopy
STORM	Stochastic Optical Reconstruction Microscopy
TEM	Transmission Electron Microscopy
TIRFM	Total Internal Reflection Fluorescence Microscopy
VMM	Viscoelastic Mapping Microscopy
